# Evidence of a Potential Nursery Area for the Great Hammerhead Shark, *Sphyrna mokarran*, in the Largest Coral Reef System of the Southwestern Atlantic

**DOI:** 10.1002/ece3.74207

**Published:** 2026-08-14

**Authors:** Jones Santander‐Neto, Rodrigo Barreto, Guilherme dos Santos Lírio, Michael Heithaus, Otto Bismarck Fazzano Gadig, Maurício Hostim‐Silva

**Affiliations:** ^1^ Laboratório de Dinâmica de Populações Marinhas – DIMAR Instituto Federal de Educação, Ciência e Tecnologia do Espírito Santo ‐ Ifes Piúma Brazil; ^2^ Laboratório de Ecologia de Peixes Marinhos – LEPMAR Universidade Federal do Espírito Santo, Rodovia Governador Mário Covas São Mateus Brazil; ^3^ Department of Biological Sciences, Institute of Environment Florida International University North Miami Florida USA; ^4^ Laboratório de Pesquisa de Elasmobrânquios – ELASMOBRASIL, Instituto de Biociências Universidade Estadual Paulista São Vicente Brazil

**Keywords:** Abrolhos Bank, conservation, early life stages, elasmobranchs, essential fish habitat, fisheries‐dependent data, *Sphyrna mokarran*

## Abstract

Identifying Essential Fish Habitats (EFHs) is fundamental for the conservation of threatened sharks, particularly those affected by overfishing and habitat degradation. The great hammerhead shark ( *Sphyrna mokarran* ), listed as Critically Endangered globally and regionally, remains poorly studied in terms of habitat use and early life ecology. Here, we present evidence suggesting that the Abrolhos Bank (the largest coral reef system in the southwestern Atlantic) may be used for early life stages of *S. mokarran* . Between April 2018 and May 2019, 58 individuals were recorded from artisanal gillnet fisheries operating in the region. Most specimens were identified as neonates or young‐of‐the‐year (YOY) based on total length and presence of umbilical scars. Kernel density estimation revealed spatially structured aggregations, with neonates concentrated in shallow reef‐associated areas. Temporal variation in catch per unit effort (CPUE) indicated recruitment pulses during late summer and early autumn, consistent with localized parturition. Although the study was limited to 1 year and subject to gear selectivity, the predominance of neonates and YOY, their persistence across consecutive months, and the absence of mature sharks suggest localized use by early life stages. These findings partially meet recognized nursery criteria and highlight the ecological significance of the Abrolhos Bank for *S. mokarran* . Protecting this coastal habitat through the expansion of marine protected area boundaries, adoption of bycatch‐reduction measures, and implementation of long‐term monitoring programs may contribute to the recovery of this imperiled species.

## Graphical abstract

We present evidence suggesting that the Abrolhos Bank functions as key habitat for early life stages of *Sphyrna mokarran* . Kernel density estimation revealed spatially structured aggregations, with neonates and young‐of‐the‐year concentrated in shallow reef‐associated areas. Temporal variation in catch per unit effort (CPUE) indicated recruitment pulses during late summer and early autumn, consistent with localized parturition.

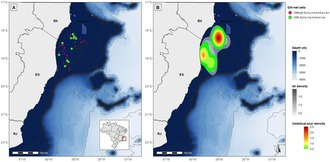

## 1 Introduction

Essential Fish Habitats (EFHs) are critical areas required for the reproduction, growth, feeding, and shelter of fish, encompassing diverse environments such as coral reefs, bays, kelp forests, and wetlands (NOAA 1996). Identifying these habitats is a key component of fisheries management and supports conservation strategies aimed at sustaining fish populations (Heupel et al. 2007). For sharks, EFH studies have predominantly focused on nursery areas. However, many of these classifications are based solely on the presence of neonates or juveniles, often without rigorous testing against established nursery criteria (Beck et al. 2001; Heupel et al. 2007). This limitation may lead to overestimation of nursery ground extent and weaken conservation outcomes (Heupel et al. 2007).

Beck et al. (2001) defined nursery areas as locations where juveniles exhibit higher abundance, reduced predation risk, and enhanced growth, thereby contributing significantly to adult populations. Heupel et al. (2007, 2019) refined these criteria for chondrichthyan fishes, specifying that neonates and young‐of‐the‐year (YOY) (1) must be relatively more abundant in a given area, (2) must demonstrate site fidelity, and (3) must consistently use the area across multiple years.

*Sphyrna mokarran* has a global distribution in tropical and warm‐temperate coastal waters, inhabiting both coastal and semi‐oceanic regions and exhibiting nomadic, seasonally migratory behavior (Rigby et al. 2019; Ebert et al. 2021). The species can exceed 550 cm in total length (TL), reaches maturity at approximately 220 cm TL, and has an 11‐month gestation period producing litters of 6 to 42 pups born at about 65 cm TL (Gilbert 1967; Stevens and Lyle 1989; Ebert et al. 2021). Individuals with presence of umbilical scar are neonates, whereas those lacking an umbilical scar but falling within the expected size range for the first year of life are considered young‐of‐the‐year (YOY), reflecting early postnatal growth. In large coastal sharks such as *S. mokarran* , this stage typically encompasses individuals with TL ranging from approximately 103 cm to over 160 cm, consistent with the observed growth variability in the species (Piercy et al. 2010). Recognizing this distinction is essential for interpreting the presence of small individuals in a given area and assessing whether such occurrences indicate parturition or juvenile residency.

Nursery areas for coastal carcharhinid sharks have been more clearly delineated due to their preference for shallow, nearshore habitats (Castro 1993; Heupel et al. 2007). However, identifying nurseries for large‐bodied, migratory species such as the great hammerhead shark ( *S. mokarran* ) remains challenging. Some studies suggest that estuarine and reef‐associated environments in the southeastern United States and the Indo‐Pacific may serve as important nursery grounds for *S. mokarran* (Drymon et al. 2010; Chin et al. 2017). Nevertheless, the species' extensive movements and wide habitat use complicate direct comparisons with more localized coastal nurseries.

The Abrolhos Bank, located off the eastern central Brazilian coast, is a region of high ecological and economic importance (Previero and Gasalla 2018). It encompasses the largest coral reef system in the South Atlantic (8844 km^2^) and features a diverse mosaic of habitats, including coral reefs, rhodolith beds (20,904 km^2^), mangroves, and unconsolidated sediments (Leão et al. 2003; Bastos et al. 2013; Moura et al. 2013). The Abrolhos Archipelago comprises five volcanic islands (Santa Bárbara, Redonda, Sueste, Guarita, and Siriba) surrounded by shallow reefs that serve as essential habitats for numerous marine species (Francini‐Filho and Moura 2008). This archipelago forms the central part of the Abrolhos National Marine Park, Brazil's first marine protected area, which has played a key role in biodiversity conservation and fisheries management (Moura et al. 2013).

The Abrolhos Bank includes both fully protected marine protected areas (MPAs) and sustainable‐use reserves, such as the Cassurubá and Corumbau Marine Extractive Reserves and the Ponta da Baleia Environmental Protection Area (Previero and Gasalla 2020). The states of Bahia and Espírito Santo, which encompass this region, provide critical habitats for *S. mokarran* (Lessa et al. 1999; Santander‐Neto et al. 2024) but have also experienced significant declines in other fish populations due to overfishing (Francini‐Filho and Moura 2008; Freitas et al. 2011; Previero and Gasalla 2020).

This study provides fishery‐dependent evidence to evaluate whether the Abrolhos Bank, particularly the Abrolhos Archipelago and its adjacent reef systems, may function as use area for early life stages of *S. mokarran* . Specifically, we analyze temporal and spatial variation in catch rates and size composition of individuals captured during standardized monitoring to test the first criterion of shark nursery definitions (higher relative abundance of early life stages in a given area) and to identify patterns consistent with potential nursery use.

## 2 Methods

### 2.1 Study Area and Sampling Effort

Sharks were caught using bottom‐set gillnets operated by artisanal commercial fisheries on the Abrolhos Bank (16°40′ to 19°40′ S, 39°10′ to 37°20′ W), encompassing the region between southern Bahia (18°08′23.4″ S) and northern Espírito Santo (19°26′06.24″ S), Brazil. Sampling was conducted from April 2018 to March 2019 during a single monthly monitoring trip aboard the same 11.4 m fishing vessel, regardless of the total number of fishing trips performed by the vessel throughout each month. Georeferenced capture data and the number of fishing sets were recorded by the onboard observer during each monitoring trip. The observer logged the precise geographical coordinates (latitude and longitude) for every set conducted, ensuring an accurate spatial representation and catch locations throughout the study period.

The same gillnet that was used in which one of the fisheries in this study consisted of nine interconnected nylon monofilament panels (nets) measuring together 5.56 km (three nautical miles) in total length and 1.8 m in height. The nets mesh size were 60 mm (measured between opposing knots). Nets were deployed once per day for approximately 11 h (range from 6:24 to 13:21). The number of deployments per trip ranged from three to eight (equivalent to effective fishing days), totaling 54 sets across 12 sampling trips.

The data analyzed here originate from regular commercial fishing operations, which were not directed toward the capture of juvenile sharks. The gillnets were designed to target a variety of demersal teleosts, and the occurrence of *S. mokarran* represents an incidental component of this multispecies fishery. This approach provides fishery‐dependent information that reflects natural species occurrence within the fishing grounds, without gear selectivity or spatial targeting for young individuals.

This monitoring represents a 1‐year standardized dataset that provides the first fishery‐dependent records of *S. mokarran* obtained with gillnets on the Abrolhos Bank. To date, there are no records of this species in the REVIZEE Program (the Brazilian government's initiative conducted from 1995 to 2006 to assess the sustainable potential of living resources in the Exclusive Economic Zone—ZEE) or in any other national fishery database derived from gillnet surveys in the southwestern Atlantic, preventing temporal or regional comparisons. The present dataset therefore constitutes a novel baseline for this Critically Endangered species in Brazilian waters.

The duration of sampling and the limited number of captures constrain the scope of inference. The absence of mark‐recapture or telemetry data also prevents evaluation of site fidelity and multi‐year use, which are essential components of the nursery‐area framework. This study is therefore limited to describing spatial and temporal patterns of occurrence and relative abundance based on fishery‐dependent observations.

### 2.2 Biological Data Collection

Upon retrieval of the nets, each specimen was identified as *Sphyrna mokarran* following the diagnostic characteristics described by Gilbert (1967) and Ebert et al. (2021). Total length (TL, cm) and interdorsal distance (ID, cm) were measured using a measuring tape with a precision of ±0.1 cm. Sex was determined by the presence or absence of claspers.

Size at birth ranges from 65 to 70 cm TL (Stevens and Lyle 1989; Harry et al. 2011). Individuals with presence of umbilical scar were classified as neonates, which represent recently born individuals. Individuals lacking an umbilical scar but falling within the size range associated with the first year of life were classified as young‐of‐the‐year (YOY). Given the observed individual growth variability for *S. mokarran* , this YOY category included specimens ranging from those lacking umbilical scar (above 65–70 cm) up to 160 cm in total length (Piercy et al. 2010).

### 2.3 Data Analysis

Histograms of total length were used to describe the size distribution of *S. mokarran* by sex and by presence or absence of an umbilical scar. All analyses and figures were performed in R version 4.2.0 (R Core Team 2022) using the packages tidyverse (Wickham et al. 2019) and ggplot2 (Wickham 2016).

Analyses were conducted to describe spatial and temporal patterns in *S. mokarran* occurrence. Because the study was based on fishery‐dependent data with limited sample size, all statistical procedures were selected to provide a descriptive and non‐inferential framework, emphasizing transparency about analytical limitations.

A chi‐square test (*χ* ^2^) was used to evaluate whether the observed sex ratio differed from the expected 1:1 proportion. Differences in total length (TL) among months were examined descriptively using boxplots and summary statistics rather than inferential tests, given the small and uneven number of individuals per month. This approach ensures consistency with the exploratory nature of the dataset and avoids overinterpretation of limited samples. Catch per unit effort (CPUE) was calculated as follows:

$$\text{CPUE}=\frac{\text{number of individuals captured}\;}{\text{number of}\;\mathrm{net}\;\text{sets}}$$

CPUE values were treated as an internal index of relative abundance within the 1‐year sampling period and were not compared with other studies, since variations in gear configuration, fishing frequency, and spatial coverage preclude standardization across datasets. Monthly variation in CPUE was examined descriptively to visualize potential temporal trends without inferring statistical significance.

Spatial analyses were conducted using georeferenced fishing locations. Bathymetric data were obtained from NOAA through the marmap package (Pante and Simon‐Bouhet 2013) and converted to a gridded data frame for visualization in ggplot2. Kernel Density Estimation (KDE) was performed to identify areas of higher relative density of captured individuals. Two KDEs were generated: one including all specimens and another restricted to individuals with presence of umbilical scars. The resulting density surfaces were overlaid to identify areas of overlap, highlighting potential parturition or early‐residency zones.

The KDEs and maps were produced using the R packages sf (v1.0–16), ggplot2 (v.3.5.2), geobr (v1.9.1), and ggspatial (v1.1.9). Base maps were created using the sf, geobr, and ggspatial packages. Official 2020 shapefiles were used to delineate coastal state boundaries, and the full polygon of the Abrolhos Bank was incorporated to represent the actual extent of the reef system rather than a single point. Bathymetric contours were shaded with a gray‐scale gradient to illustrate depth variation. The color scales in all maps were standardized to express relative density only, without implying quantitative abundance. The KDE outputs are therefore intended purely for visualization of spatial patterns, not for inference of population size or absolute distribution.

## 3 Results

A total of 58 individuals of the great hammerhead shark *S. mokarran* were recorded from 14 of the 54 monitored gillnet sets, which were distributed across seven of the 12 total fishing trips conducted between April 2018 and March 2019. In the remaining five trips, no *S. mokarran* were recorded. Notably, all 12 fishing trips included sets where the species was absent, reflecting the opportunistic nature of the fishery‐dependent sampling. Total length (TL) ranged from 71.5 to 165 cm, with a mean of 104.6 ± 18.8 cm. Of all individuals measured, 31 were males (mean TL = 103.5 ± 20.2 cm, range: 76.5–165 cm) and 24 were females (mean TL = 106.0 ± 17.3 cm, range: 71.5–131 cm), while three lacked sex and size information. The sex ratio did not deviate significantly from parity (χ^2^ = 0.89, *p* = 0.42), and no mature specimens were recorded throughout the study period.

**FIGURE 1:**
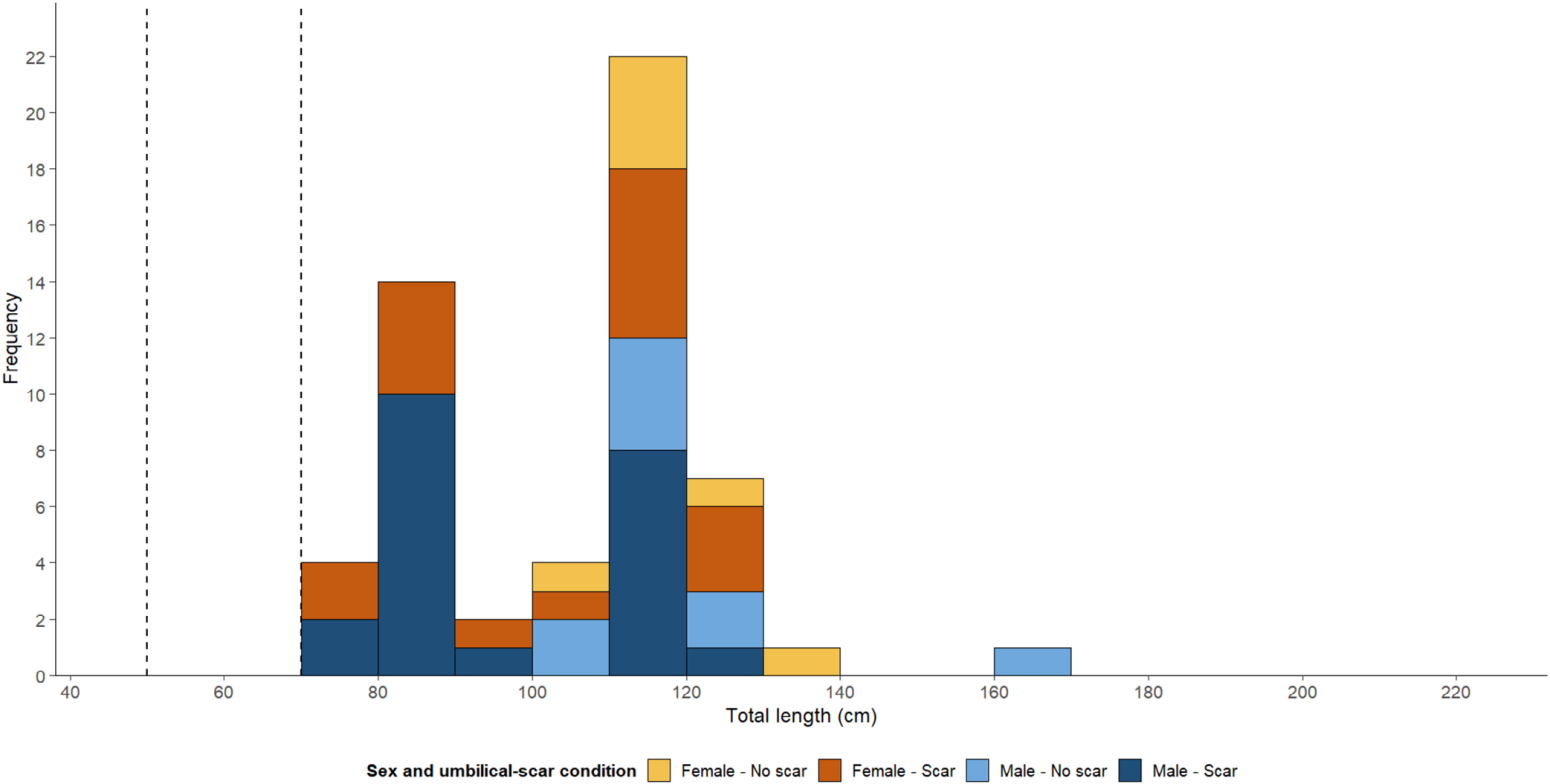
Size‐frequency distribution of *Sphyrna mokarran* by sex and umbilical‐scar condition. Vertical dashed lines delimit the reported total length at birth (50–70 cm). Bars are stacked by sex and umbilical‐scar condition. Female individuals are shown in yellow‐to‐orange tones, whereas males are shown in blue tones. Within each sex, darker shades indicate individuals with an umbilical scar, while lighter shades indicate individuals lacking an umbilical scar.

The size‐frequency distribution of *S. mokarran* by sex and umbilical‐scar condition indicates that most individuals measured between 80 and 120 cm TL, and the overall size structure was unimodal, dominated by early life stages (neonates and YOY) (Figure 1). The length composition confirms that all captured individuals were immature, composed predominantly of neonates and YOY. Individuals with presence of umbilical scar (*n* = 39) averaged 98.6 ± 17.4 cm TL, whereas those without scars (*n* = 19) averaged 119.0 ± 13.8 cm TL. This distinction suggests a transitional size range around 110–120 cm TL, consistent with postnatal growth within the first year of life.

**FIGURE 2:**
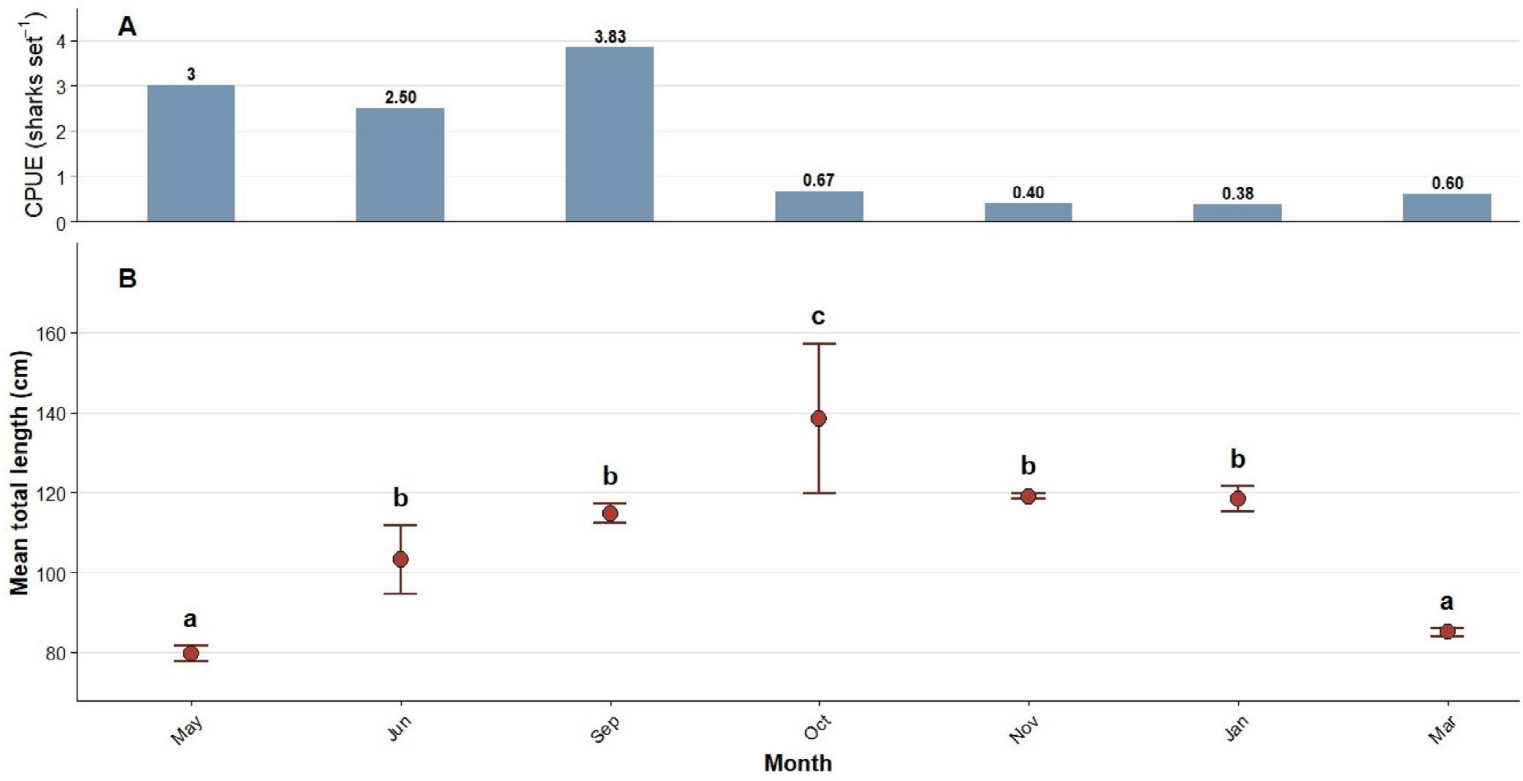
Monthly variation in catch per unit effort (CPUE) and body size of *Sphyrna mokarran* captured between April 2018 and March 2019. (A) Monthly CPUE expressed as the number of sharks captured per gill‐net set. (B) Mean total length of captured individuals by month, with error bars representing the lower and upper confidence limits. Boxed lowercase letters indicate post hoc statistical groups; months sharing the same letter do not differ significantly (*p* ≥ 0.05), whereas months with different letters differ significantly (*p* < 0.05).

Monthly variation in catch per unit effort (CPUE, individuals per set) fluctuated across the 1‐year sampling window, with higher values recorded in May, June, and September 2018. Mean total length (TL) varied among months, ranging from approximately 90 cm in May 2018 to 140 cm in October 2018. Differences in TL among months were statistically significant (Kruskal–Wallis test, *p* < 0.05), although the number of individuals recorded per month was low (Figure 2).

**FIGURE 3:**
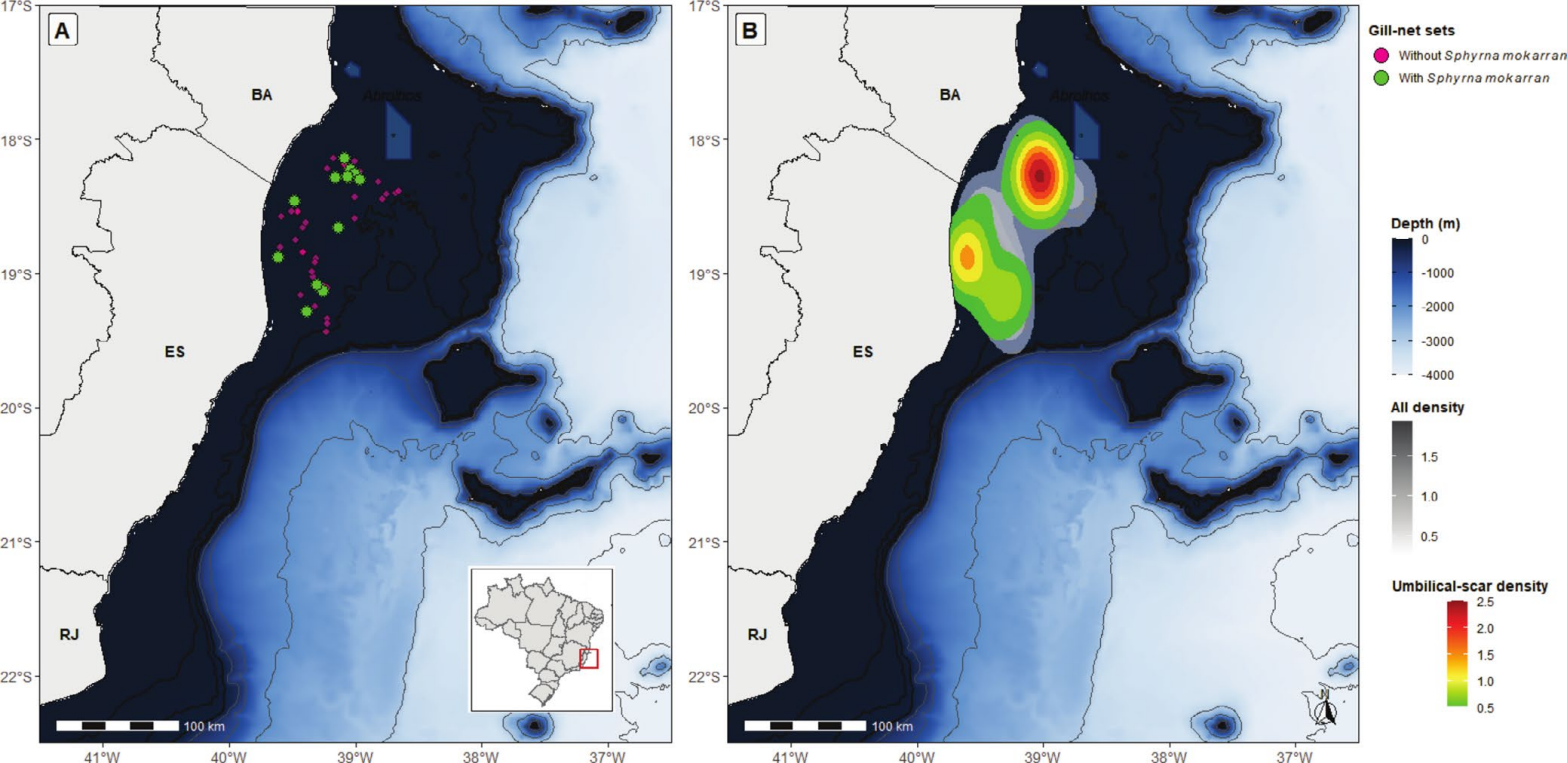
Spatial distribution and kernel density of *Sphyrna mokarran* captures in the Abrolhos Bank region, eastern Brazil. (A) Gill‐net sets with (green) and without (pink) *S. mokarran* captures plotted over shaded bathymetry; the inset shows the location of the study area in Brazil. (B) Overlaid kernel density estimates (KDEs) showing the relative spatial density of all captured individuals (gray scale) and of individuals with umbilical scars (green–yellow–orange–red). Warmer colors indicate higher relative density. KDE values are relative and should be interpreted as indicators of spatial clustering rather than absolute abundance. The blue polygon delineates the Abrolhos Marine Protected Area. Lighter blues indicate deeper waters and darker blues indicate shallower waters.

Spatially, capture locations were concentrated along the inner and mid‐shelf of southern Bahia and northern Espírito Santo (Figure 3). The KDE maps reveal two areas of higher encounter frequency: one adjacent to the Abrolhos Reef Complex and another along the mid‐shelf region off northern Espírito Santo. The density gradient expresses relative clustering of occurrences within the surveyed area (Figure 3) rather than absolute abundance, and the bathymetric shading indicates the spatial extent of shallow shelf habitats (< 50 m depth) where captures were concentrated.

Overall, the dataset documents recurrent occurrences of neonates and YOY of *S. mokarran* within the Abrolhos Bank region during the sampling period, with no records of mature individuals. These findings indicate that the surveyed area functions, at least temporarily, as habitat for early life stages of the species within a shallow coastal environment of the southwestern Atlantic. Given the limited temporal and numerical scope of the data, the results are interpreted as exploratory evidence of habitat use by neonates and YOY, providing an ecological basis for future standardized assessments on early‐stage distribution and habitat function in the southwestern Atlantic.

## 4 Discussion

Studies on hammerhead sharks based on fisheries‐dependent data are inherently challenging because multiple Sphyrna species are frequently grouped under the generic category “hammerhead sharks,” leading to misidentification and taxonomic ambiguity (Barreto et al. 2016; Bezerra et al. 2016; Gallagher and Klimley 2018). Landings are typically dominated by *Sphyrna lewini* , further complicating assessments focused specifically on *S. mokarran* . Despite these limitations, the present dataset represents one of the largest verified coastal samples of *S. mokarran* reported for Brazil, exceeding previous records derived from research surveys and commercial fisheries (Lessa 1986; Meneses et al. 2011; Santander‐Neto and Faria 2020; Oliveira‐Junior et al. 2022; Pinheiro et al. 2023).

Although some studies have reported the presence of neonates and YOY of *S. mokarran* , specific areas where these individuals consistently occur have rarely been identified. Neonates are often recorded over broad spatial extents and in association with individuals at other developmental stages (Stevens and Lyle 1989; Cliff 1995; Harry et al. 2011; Pinheiro et al. 2023), making it difficult to distinguish localized nursery use from general habitat overlap. In this context, the predominance of neonates and YOY observed in the Abrolhos Bank is relatively uncommon when compared with the existing literature. Nevertheless, this pattern should be interpreted with caution, as the 1‐year temporal scale of the study and the selectivity of gillnets may have favored the capture of smaller individuals. Accordingly, these findings are best interpreted as preliminary evidence of local use by early life stages rather than conclusive support for nursery function.

The size composition indicated that none of the captured individuals reached the estimated size at sexual maturity for either sex, supporting the inference that the sampled population consisted primarily of immature individuals. The balanced sex ratio aligns with embryonic expectations and with patterns reported for *Sphyrna mokarran* in northern Australia (Stevens and Lyle 1989) and in other regions (Cliff 1995; Piercy et al. 2010; Harry et al. 2011). However, departures from parity have been documented elsewhere, including male‐biased ratios in northeastern Brazil (Pinheiro et al. 2023) and female‐biased ratios in French Polynesia (Boube et al. 2023), suggesting that sexual segregation may occur depending on local environmental or behavioral factors. Minor differences in size distributions between sexes may reflect early habitat partitioning or growth divergence, but monthly sample sizes limit inference.

Temporal patterns in catch per unit effort (CPUE) and total length (TL) revealed pulses of small individuals during late autumn and early spring. These increases likely reflect episodic recruitment or transient aggregations rather than stable seasonal peaks, given the uneven sampling effort among months. Mean TL values remained consistently within the size range of immature individuals throughout the study period, without evidence of the regular presence of larger juveniles or adults.

The occurrence of neonates with presence of umbilical scars indicates recent parturition. Based on regional context, this finding is consistent with pupping seasons reported for *Sphyrna mokarran* in tropical and subtropical areas, which generally occur during the months corresponding to February to May in the southwestern Atlantic (Fourmanoir 1961; Clark and Von Schmidt 1965; Cadenat and Blache 1981; Stevens and Lyle 1989; Harry et al. 2011). Importantly, the temporal window of parturition inferred from neonatal presence does not necessarily coincide with the months of highest CPUE or smallest mean sizes observed in this study.

Although CPUE was used strictly as an internal index of relative occurrence, the repeated detection of neonates and YOY across consecutive months suggests short‐term retention or repeated use of the same coastal zone. Furthermore, neonates and YOY were recorded starting in May 2018, as well as in January and March 2019, suggesting a temporal pattern in the occurrence of early life stages. This potential for recurrent use of the area as a parturition site is further supported by recent evidence, including the capture of a pregnant female with near‐term embryos in January 2026 off the coast of Linhares, within the Abrolhos Bank region. This event was reported due to the species' threatened status (personal communication). In this sense, the observed pattern provides partial support for the second criterion proposed by Heupel et al. (2007), namely the tendency of early life stages to remain in or repeatedly use a specific area. However, given the absence of multi‐year data and individual‐based movement information, these findings should be interpreted as preliminary evidence of localized juvenile use rather than definitive confirmation of nursery function.

Spatial analyses using kernel density estimation (KDE) identified two main clusters of individuals: a high‐density hotspot on the southern Abrolhos shelf dominated by neonates and a secondary aggregation off Espírito Santo. The KDE overlays results for the full dataset and for neonates alone, with bathymetric contours and the Abrolhos polygon delineated. These density peaks indicate spatially structured occurrences of neonates and young‐of‐the‐year, suggesting localized parturition or early postnatal retention. The brief persistence of umbilical scars further supports the interpretation that these aggregations reflect recent birth events.

Together, these findings satisfy the first criterion proposed by Heupel et al. (2007), namely a higher relative abundance of neonates and YOY within a defined area, and are consistent with the refined framework of Heupel et al. (2019), which emphasizes demographic relevance rather than occurrence alone. Although repeated interannual use cannot be formally demonstrated in the absence of multi‐year datasets, the sampling period encompassed consecutive months distributed across two calendar years, during which recurring pulses of YOY and neonates were observed. This pattern is compatible with short‐term reoccupation or repeated use of the area, but does not allow confirmation of long‐term site fidelity. Following the conservative approach adopted by Chiriboga‐Paredes et al. (2022) for *Sphyrna lewini* in the Galápagos Islands, we therefore describe the Abrolhos Bank as a putative nursery area pending further verification. Long‐term monitoring incorporating tagging, telemetry, and genetic approaches will be required to assess whether females consistently return to this region to give birth.

Several limitations must be acknowledged. Gear selectivity likely biased catches toward smaller individuals, and monthly sample sizes were insufficient for robust statistical comparisons. The single‐year duration limits our capacity to test interannual fidelity, and the absence of environmental covariates or behavioral data restricts mechanistic inference. Future studies should integrate spatiotemporal modeling, telemetry, and habitat characterization to evaluate nursery persistence and drivers of habitat use.

From a conservation perspective, the Abrolhos Bank holds exceptional ecological value and is recognized as an Important Shark and Ray Area (Jabado et al. 2025). The neonatal hotspots identified here overlap with zones of active coastal fishing, indicating that early life stages are exposed to bycatch risk. In contrast, adults are widely captured by industrial pelagic longline fleets targeting tunas, billfishes, and other sharks along the Brazilian Exclusive Economic Zone (Barreto et al. 2016), meaning that all life stages of this Critically Endangered species are vulnerable to fisheries mortality. Expanding Marine Protected Area (MPA) boundaries to encompass these neonatal hotspots could strengthen protection of essential habitats. Such actions should be combined with seasonal closures during the parturition period, bycatch‐reduction measures for artisanal fisheries, and long‐term programs integrating genetic, isotopic, and eDNA tools to verify interannual use and connectivity with offshore populations.

Overall, the convergence of biological, temporal, and spatial evidence indicates that the Abrolhos Bank fulfills several elements of the shark nursery framework, even if it cannot yet be classified as a confirmed nursery sensu Heupel et al. (2007, 2019). The consistent occurrence of neonates and YOY in shallow coral‐associated habitats, combined with the absence of adults, highlights the area's ecological importance for early development. Designating this region as a putative nursery is therefore appropriate and aligns with a precautionary conservation approach. Protecting these critical habitats is essential to mitigate cumulative fishing pressure across all life stages and to support the recovery of *Sphyrna mokarran* populations in the southwestern Atlantic.

## Author Contributions

**Jones Santander‐Neto:** conceptualization (equal), data curation (equal), formal analysis (equal), methodology (equal), project administration (lead), resources (equal), software (supporting), supervision (lead), visualization (equal), writing – original draft (lead), writing – review and editing (equal). **Rodrigo Barreto:** data curation (equal), formal analysis (equal), methodology (equal), software (lead), validation (equal), visualization (equal), writing – review and editing (equal). **Guilherme dos Santos Lírio:** data curation (equal), investigation (equal), methodology (supporting), resources (equal), writing – review and editing (equal). **Michael Heithaus:** validation (equal), visualization (equal), writing – review and editing (equal). **Otto Bismarck Fazzano Gadig:** conceptualization (equal), visualization (equal), writing – review and editing (equal). **Maurício Hostim‐Silva:** conceptualization (equal), project administration (supporting), supervision (supporting), validation (equal), writing – review and editing (equal).

## Conflicts of Interest

The authors declare no conflicts of interest.

## Data Availability

Data is available on: https://zenodo.org/records/21762252.
